# Functional Outcomes of Bone-Patellar Tendon-Bone Versus Quadrupled Semitendinosus and Gracilis Autografts for Anterior Cruciate Ligament Reconstruction

**DOI:** 10.7759/cureus.66945

**Published:** 2024-08-15

**Authors:** Mohammed Inayathulla Khan, Inas Ismail, Savith Shetty, Jithin A Jebbar, Afra Farheen Faiaz, Shameez Mohammed, Abhishek V Shetty, Imthiaz Ahammed, Mohammed Shahid

**Affiliations:** 1 Orthopedics, Yenepoya Medical College and Hospital, Mangalore, IND; 2 Anesthesiology, Kanachur Institute of Medical Sciences, Mangalore, IND; 3 Orthopedic Surgery, Yenepoya Medical College and Hospital, Mangalore, IND; 4 Orthopedics, Vydehi Institute of Medical Sciences and Research Centre, Bangalore, IND

**Keywords:** anterior cruciate ligament, autograft, bone-patellar tendon-bone, gracilis, semitendinosus

## Abstract

Introduction

Anterior cruciate ligament reconstruction (ACLR) with autografts has been available for decades; however, the choice of graft is still debated. Here, we compared the functional outcomes of the two most widely used autografts, bone-patella tendon-bone (BPTB) and quadruple-stranded semitendinosus/gracilis (ST/G) autografts, at six months following ACLR.

Materials and methods

This prospective study was performed in the Department of Orthopedics of Yenepoya Medical College and Hospital located in Mangalore, Karnataka, India, a tertiary care institute over a period of 18 months (November 2018 to April 2020). The study included 38 adult patients who underwent ACLR and were randomly divided into two groups: BPTB autograft (n=19) or ST/G autograft (N=19). The patients were followed up at one-, three-, and six months. Postoperatively, surgical morbidity, knee stability functional outcome on Lysholm score, and knee range of motion (ROM) were assessed.

Results

The groups were homogenous and comparable regarding age, sex, side of ACL affected, duration of tear to treatment, and muscle wasting (all p-values > 0.05). At six months, the majority of the patients had a tibial translation of 0-2 mm on the Lachman test and anterior drawer test, and the groups did not differ significantly (both p-values > 0.05). Additionally, at six months, both groups demonstrated a significant increase in mean Lysholm score and mean ROM (both p-values < 0.001). However, the groups did not differ in mean Lysholm score and mean ROM at baseline and any of the follow-up visits (all p-values > 0.05).

Conclusion

At six months, ACLR with BPTB and ST/G autografts produced significant and comparable knee stability, functional outcome, and ROM.

## Introduction

Injury of the anterior cruciate ligament (ACL) is frequently observed, especially among physically active young individuals, and can lead to functional impairment [1]. Though ACL injuries may be contact or non-contact, they are predominantly a result of a non-contact mechanism [2]. In general and in the sports active population, the annual incidence of unilateral ACL tear varies from 0.01-0.08% and 1.5-1.7%, respectively. ACL tear produces knee instability, resulting in the symptom of giving way and a higher risk of meniscal injury [3]. Additionally, the lifetime risk of osteoarthritis knee is increased when the ACL and meniscal injuries co-exist [4].

The goal of ACL tear management is to restore knee stability and prevent the early onset of osteoarthritis due to joint instability [3]. Considering the societal and economic impact of ACL tears, ACL reconstruction (ACLR) is the preferred cost-effective treatment strategy compared to rehabilitation [1]. 

For ACLR, bone-patellar tendon-bone (BPTB) and hamstring tendon (HST) autografts are the predominant grafts employed. Both BPTB and HST autografts are reported to have identical outcomes [5]. However, relative to HST grafts, BPTB grafts have a lower risk of failure and are more stable, as well as being considered the preferred graft in young and active individuals. While BPTB grafts are demonstrated to be associated with significantly higher knee pain relative to HST grafts [6]. Though various studies have compared BPTB autograft with quadruple graft of semitendinosus and gracilis (ST/G) tendons, they have not been sufficiently evaluated in Indian patients with ACL tears. Thus, we compared the functional outcomes of arthroscopically assisted ACLR using BPTB and quadruple-stranded ST/G grafts in terms of postoperative knee stability, subjective knee functions, graft site morbidity, and range of motion (ROM).

## Materials and methods

This prospective and comparative study was performed in the Department of Orthopedics of Yenepoya Medical College and Hospital located in Mangalore, Karnataka, India, a tertiary care institute over a period of 18 months (November 2019 to April 2020). The study included adult patients aged 18 - 40 years of either sex, presenting with clinically and radiologically diagnosed acute ACL tear (within the past six months) and involving intra-substance tear with femoral avulsion. While patients with systemic illness compromising their pre-anesthetic fitness, bilateral ACL tear, multi-ligament injuries, ACL tear associated with tibial spine avulsion, articular cartilage lesions > Grade II of the International Cartilage Repair Society, and previous injuries/surgeries on the affected knee were excluded. The study commenced after approval of the protocol by the Institutional Ethics Committee and obtaining written informed consent from the patients.

A total of 38 patients were randomly and equally divided into two groups: BPTB autograft (n=19) or ST/G autograft (n=19). In each group, the same surgeon performed arthroscopic ACLR on all the patients using the same technique. Randomization was performed on the day of surgery with an odd and even number of enrolled patients receiving BPTB and ST/G autografts, respectively. Pre-operatively, data related to the age, sex, side of the knee affected, duration and type of ACL tear, associated injuries, presence of muscle wasting, and type of femoral fixation used were recorded. All the patients were started on postoperative ACL protocol, adapted from Wilk et al. [7]. The patients were followed up at one-, three-, and six months. Postoperatively, surgical morbidity, functional outcome on Lysholm score, and knee ROM were assessed. Additionally, X-rays were taken to check the position of the endobutton and interference screws (ISs). At six months, knee stability was assessed with the Lachman test and the anterior drawer test. The data was analyzed with IBM SPSS Statistics for Windows, Version 23 (Released 2015; IBM Corp., Armonk, New York, United States) for Windows. The categorical and continuous variables are represented as frequency (percentage) and mean (standard deviation, SD), respectively. Between the groups, comparisons of categorical and continuous variables were performed with chi-square and independent sample t-tests, respectively. Moreover, within-group comparison of continuous variables was performed with repeated measures analysis of variance (ANOVA) followed by post-hoc analysis with Bonferroni’s multiple comparison test. A two-tailed probability value (p-value) of less than 0.05 was considered statistically significant.

## Results

The BPTB group had a higher mean age relative to the ST/G group; however, there was no statistically significant difference (p-value=0.078). In both the groups, male gender and right-sided ACL were predominantly affected (p-value=0.547 and 0.732, respectively). Additionally, the duration of tear to treatment was comparable between the groups (p-value=0.673). In the BPTB group, isolated ACL tear was most common, while ACL + medial meniscus (MM) was most frequently affected in the ST/G group, and both reached statistical significance (p-value=0.023 and 0.017, respectively). However, the groups were comparable in terms of the involvement of ACL + lateral meniscus (LM) (p-value=1.000) and ACL + LM + MM (p-value=0.631). Further assessment suggested that most of the patients in both groups had no associated injuries with no significant difference between the groups (p-value=0.051). Though the groups did not differ in any of the associated injuries (all p-values > 0.05), the ST/G groups had significantly higher involvement of MM relative to the BPTB group (p-value=0.017). Both the groups had higher involvement of patients with muscle wasting; however, they did not differ significantly (p-value > 0.05). Significantly greater proportion of patients in the ST/G group underwent adjustable loop (AL) (p-value=0.005) and FL femoral fixation (p-value=0.008), while significantly greater proportion of patients in the BPTB group required IS for femoral fixation (p-value < 0.0001) (Table 1).

**Table 1:** Baseline and clinical characteristics BPTB: bone-patellar tendon-bone; ST/G: semitendinosus/gracilis; ACL: anterior cruciate ligament; MCL: medial collateral ligament; MM: medial meniscus; LM: lateral meniscus; AL: adjustable loop; FL: fixed loop; IS: interference screw

Characteristics	BPTB group (n=19)	ST/G group (n=19)	p-value
Age, year, mean ± SD	27.95 ± 8.50	23.58 ± 6.19	0.078
Sex, Male, n (%)	18 (94.7%)	17 (89.5%)	0.547
Side involved, Right, n (%)	12 (63.2%)	13 (68.4%)	0.732
Duration of tear, months, mean ± SD	2.63 ± 2.57	2.37 ± 0.83	0.673
Type of ACL tear, n (%)			
Isolated ACL	13 (68.42%)	6 (31.58%)	0.023
ACL + MM	1 (5.26%)	7 (36.84%)	0.017
ACL + LM	3 (15.79%)	3 (15.79%)	1.000
ACL + LM + MM	2 (10.53%)	3 (15.79%)	0.631
Associated injuries, n (%)			
None	13 (68.42%)	7 (36.84%)	0.051
LM	2 (10.53%)	2 (10.53%)	1.000
LM + MCL	1 (5.26%)	1 (5.26%)	1.000
MCL	1 (5.26%)	0 (0%)	0.311
MM	1 (5.26%)	7 (36.84%)	0.017
MM + LM	1 (5.26%)	1 (5.26%)	1.000
MM + MCL	0 (0%)	1 (5.26%)	0.311
Muscle wasting, n (%)	17 (89.47%)	18 (94.74%)	0.547
Femoral fixation, n (%)			
AL	2 (10.53%)	10 (52.63%)	0.005
FL	0 (0%)	6 (31.58%)	0.008
IS	17 (89.47%)	3 (15.79%)	< 0.0001

**Figure 1:**
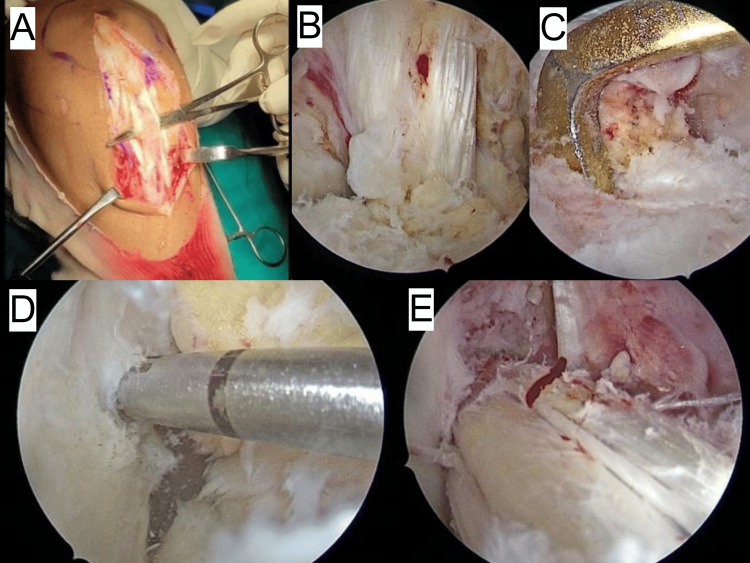
Intraoperative images of ACLR using BPTB graft (Case 1) A: identifying and exposure of the patellar tendon; B: preparation of the tibial footprint; C: placement of the tibial jig; D: preparation of the femoral tunnel; E: bone-patellar tendon-bone graft placed inside the tunnels ACLR: anterior cruciate ligament reconstruction; BPTB: bone-patella tendon-bone

**Figure 2:**
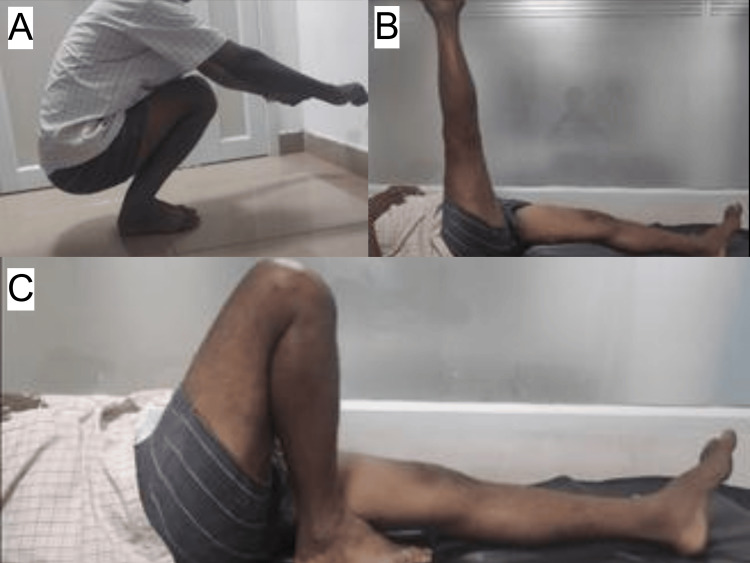
Six months follow-up ROM of ACLR using BPTB graft A: patient able to squat; B: patient able to perform SLR up-to 90 degrees; C: patient able to perform full ROM ACLR: anterior cruciate ligament reconstruction; BPTB: bone-patella tendon-bone; ROM: range of motion; SLR: straight leg raise

**Figure 3:**
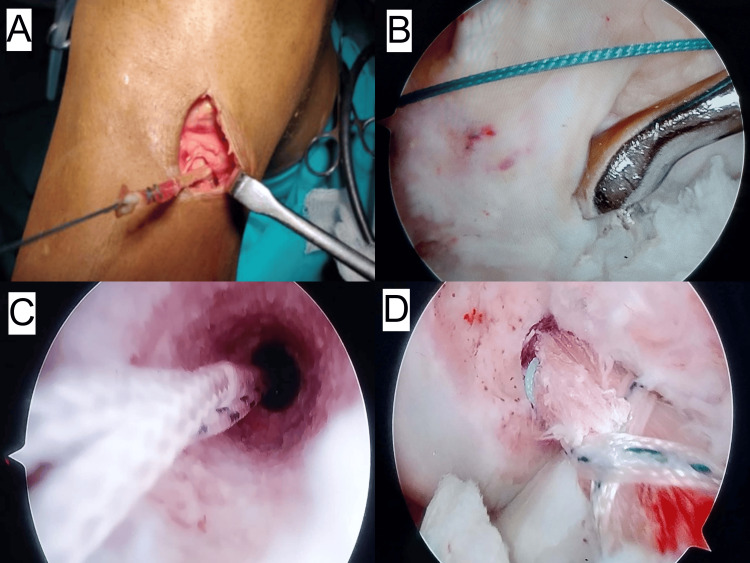
Intraoperative images of ACLR using ST/G graft A: semitendinosus/gracilis (ST/G) graft harvesting; B: tibial tunnel preparation; C: femoral tunnel preparation; D: graft secured in place ACLR: anterior cruciate ligament reconstruction

**Figure 4:**
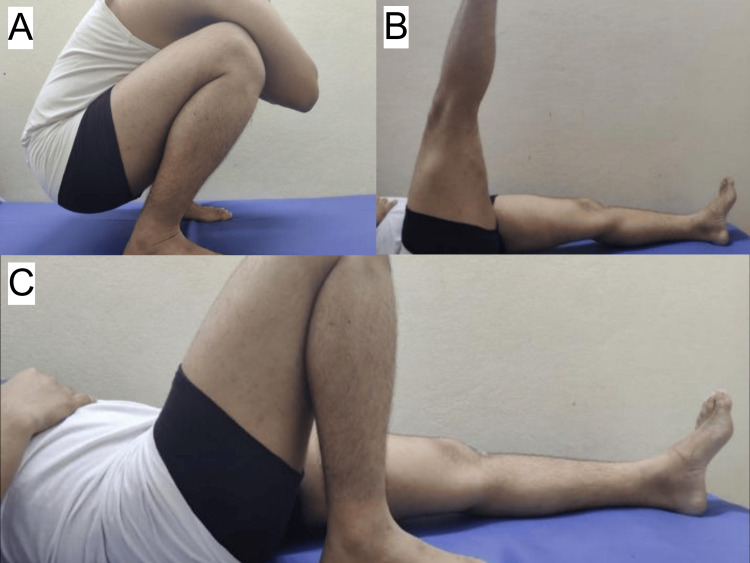
Six months follow-up ROM of ACLR using ST/G graft A: patient able to squat; B: patient able to perform SLR up-to 90 degrees; C: patient able to perform full ROM ACLR: anterior cruciate ligament reconstruction; ROM: range of motion; SLR: straight leg raise

During the follow-up period, one patient in each group developed infection (surgical site infection in the BPTB group and infection with effusion in the ST/G group), and the groups were comparable in terms of the postoperative infection (all p-values > 0.05). At six months, the majority of the patients had a tibial translation of 0-2 mm on the Lachman test and anterior drawer test, and the groups did not differ significantly (both p-values > 0.05). Additionally, in both groups, post-hoc analysis revealed significant improvement in mean Lysholm score and mean knee ROM from baseline to various follow-up visits and between various follow-up visits (all p-values < 0.001) (Figures 1-4).

However, the groups did not differ in mean Lysholm score and mean knee ROM at baseline and any of the follow-up visits (all p-values > 0.05) (Table 2).

**Table 2:** Outcome characteristics *from baseline to six months; #from one month to six months; BPTB: bone-patellar tendon-bone; ST/G: semitendinosus-gracilis; ROM: range of motion

Outcome characteristics	BPTB group (n=19)	ST/G group (n=19)	p-value
Infection, n (%)			
None	18 (94.74%)	18 (94.74%)	1.000
Infection with effusion	0 (0%)	1 (5.26%)	0.311
Surgical site infection	1 (5.26%)	0 (0%)	
Lachman test, n (%)			0.631
0-2 mm	16 (84.21%)	17 (89.47%)	
3-5 mm	3 (15.79%)	2 (10.53%)	
Anterior drawer test, n (%)			0.631
0-2 mm	16 (84.21%)	17 (89.47%)	
3-5 mm	3 (15.79%)	2 (10.53%)	
Lysholm score, mean ± SD			
Baseline	48.37 ± 7.39	47.47 ± 6.83	0.700
1-month	60.37 ± 7.52	59.26 ± 7.72	0.657
3-month	72 ± 5.98	74.63 ± 6.14	0.189
6-month	86.05 ± 5.3	87.68 ± 5.23	0.346
Change*	37.68 ± 6.15	40.21 ± 7.66	0.270
p-value	< 0.001	< 0.001	
Knee ROM, mean ± SD			
1-month	88.95 ± 3.15	89.47 ± 2.29	0.560
3-month	106.32 ± 9.55	109.47 ± 9.7	0.319
6-month	113.16 ± 7.49	113.68 ± 7.61	0.831
Change^#^	24.21 ± 6.93	24.21 ± 6.07	1.000
p-value	< 0.001	< 0.001	

## Discussion

The principal findings of the present study suggest that both BPTB and ST/G autografts produce significant improvement in functional outcome and ROM at the end of six months. However, BPTB and ST/G autografts did not differ in functional outcome and ROM at any of the follow-up visits. Additionally, both autografts had comparable surgical morbidity. Similar findings were reported by other studies [8-10]. In randomized control trials (RCTs), Sajovic et al. presented the comparative data of five- and 11-year follow-up and demonstrated no significant difference between the BPTB and ST/G autografts in functional outcome, based on Lysholm score [9,10].

In the present study, ROM was assessed to evaluate any deficit in extension and flexion, and it was observed that both BPTB and ST/G autografts resulted in sequential and statistically significant increases in ROM over the study period, but the grafts led to comparable ROM. Other studies have found similar findings [8,11]. In an RCT, Maletis et al. showed that BPTB and ST/G autografts produced comparable ROM [8].

In the present study, evaluation of knee stability with the Lachman test and the anterior drawer test revealed comparable outcomes with both the grafts. Barenius et al. observed similar findings [11]. However, Biau et al. performed a meta-analysis and observed that the BPTB autograft provides superior stability based on the pivot shift test, but stability did not differ based on the Lachman test, which is contrary to the findings of the present study [12]. This variation could be ascribed to the size effect of the graft.

In the present study, none of the patients developed contralateral tears. However, the available literature suggests a higher contralateral tear rate with BPTB autografts relative to HST autografts [13-15]. Additionally, there was absence of graft failure in the present study. However, Arida et al. reported significantly greater graft failures in the HST graft relative to the BPTB graft [16].

In the present study, a significantly greater proportion of patients in the BPTB group had anterior knee joint pain. Contrarily, with the use of ST/G autograft, fewer donor-site complications have been reported, although it may lead to a potential hamstring muscle weakness. However, this was comparable to the findings of a meta-analysis done by Li et al., who suggest significantly less anterior knee pain and kneeling pain with the HST autograft following ACLR [17]. Regarding superficial wound infection, one patient in each group in the present study had postoperative wound infection, which subsided on initiation of the culture-based oral antibiotics; the findings are similar to those reported by Rathore et al. [18]. However, other complications of ACLR, including kneeling pain, giving away sensation in the knee, extension deficit, and patellar fracture, were not observed in any of the patients.

The present study had certain limitations. First, due to the short follow-up duration, long-term outcomes could not be evaluated. Second, though the postoperative rehabilitation protocol was identical for all the patients, its quality and consistency could not be controlled due to a lack of direct daily supervision. Third, the data pertaining to postoperative muscle strength was not collected. Finally, the effect of the treatment of associated intra-articular injuries on the study outcomes was not evaluated.

## Conclusions

At six months, ACLR with either BPTB or ST/G autografts produced improved and comparable knee stability, functional outcome, and ROM. Though anterior knee pain was significantly greater with BPTB autografts, surgical site complications were comparable with both the autografts. There was absence of graft retear or contralateral ACL tear during the study period. However, further studies with long-term follow-up are required to compare the BPTB and ST/G autografts.
